# Data on the expression and role of TREM-1 in the development of in-stent restenosis

**DOI:** 10.1016/j.dib.2017.11.065

**Published:** 2017-11-22

**Authors:** Fang Wang, Chang Li, Feng Hua Ding, Ying Shen, Jie Gao, Zhu Hui Liu, Jia Wei Chen, Rui Yan Zhang, Wei Feng Shen, Xiao Qun Wang, Lin Lu

**Affiliations:** a Department of Cardiology, RuiJin Hospital, Shanghai Jiao-Tong University School of Medicine, Shanghai, PR China; b Institute of Cardiovascular Diseases, Shanghai Jiao-Tong University School of Medicine, Shanghai, PR China

## Abstract

The data presented in this article are related to the research article entitled “Increased serum TREM-1 level is associated with in-stent restenosis, and activation of TREM-1 promotes inflammation, proliferation and migration in vascular smooth muscle cells” (Wang et al., 2017) [1], which demonstrated that TREM-1 is expressed on vascular smooth cells (VSMCs) and promotes inflammation, proliferation and migration in cultured VSMCs. In this dataset, the expression of TREM-1 in leukocytes and endothelial cells of carotid artery after ligation was evaluated. The effect of TREM-1 on stenosis was analyzed in cultured human saphenous veins (HSVs) that spontaneously undergo remodeling which involves VSMC proliferation and migration.

**Specifications Table**

**Table:** 

Subject area	Biology
More specific subject area	Vascular biology
Type of data	Graph, figure
How data was acquired	Immunofluorescence and tissue culture
Data format	Raw, analyzed
Experimental factors	Expression pattern of TREM-1 in the ligated carotid artery was detected and the thickness of cultured human saphenous vein was measured after exposure to a TREM-1-specific antagonist (LP17) or agonist (monoclonal activating antibody)
Experimental features	Both the expression and molecular function of TREM-1 in the model of vascular stenosis was analyzed
Data accessibility	The data are available with this article

**Value of the data**

• The data analyze changes in the expression of TREM-1 during the development of vascular stenosis.
• The data delineate the cellular source of TREM-1 in the vascular wall under basal conditions and after stenosis.
• The data provide evidence for the participation of TREM-1 signaling in the stenotic process.

## 1 Data

**Fig. 1:**
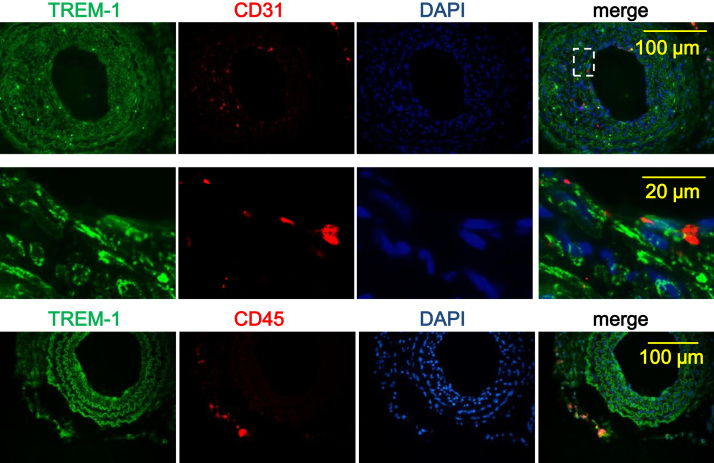
Infiltrated leukocytes but not endothelial cells express TREM-1 in the ligated carotid artery. Left common carotid arteries of C57BL/6 mice were isolated 21 days after ligation. Expression of TREM-1 in the injured vessels was determined by immunofluorescence (IF) analysis. Endothelial cells and infiltrated leukocytes were detected by staining of CD31 and CD45, respectively. Merge shows evident co-localization of TREM-1 with CD45 but not with CD31. Shown are typical images from 4 independent experiments.

**Fig. 2:**
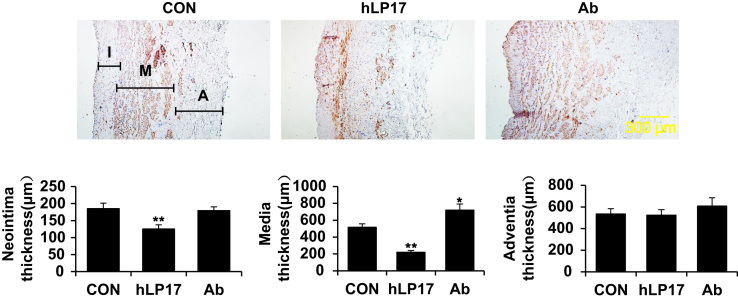
Medial thickening of the cultured human saphenous vein is modulated by TREM-1 signaling. Human saphenous vein (HSV) explants were subjected to *ex vivo* culture for 7 days with or without a synthesized human TREM-1 inhibitory peptide (hLP17) or a monoclonal TREM-1-specific activating antibody (Ab). (A) Shown are representative images of HSV sections with immunostaining for α-smooth muscle actin (brown). (B) Quantification of intimal, medial and adventitial thickness. Data are expressed as mean±SD of 4 independent experiments. *p<0.05, **p<0.01 versus control.

Dual immunofluorescence investigated whether TREM-1 was co-localized with endothelial cells (CD31^+^) or infiltrated leukocytes (CD45^+^) besides smooth muscle cells [1] within the vessel wall of the ligated carotid arteries (Fig. 1). Thickness of the intimal, medial and adventitial layers of the HSVs was measured after cultured *ex vivo* for 7 days with or without LP17 or TREM-1-activating antibody (Fig. 2).

## 2 Experimental design, materials and methods

### 2.1 Animals and carotid ligation

All animal experiments were conducted in accordance with experimental protocols approved by the Committee on Animal Resources of Shanghai Jiaotong University. Wild type C57BL/6 mice were obtained from Beijing Vital River Laboratory Animal Technology Co., Ltd (Beijing, China). Complete carotid ligation was performed on C57BL/6 mice anesthetized using 2.0% isoflurane, placed on a heated surgical board. A midline cervical incision was made and the left common carotid was isolated and ligated.

### 2.2 Immunofluorescence

Immunofluorescence was performed as previously described [2]. Paraffin-embedded sections of the carotid artery were deparaffinized and incubated with primary goat anti-TREM-1 antibody (#sc-19312, Santa Cruz Biotechnology, Dallas, TX, USA), rabbit anti-PECAM-1 antibody (#sc-1506R, Santa Cruz Biotechnology, Dallas, TX, USA), or rabbit anti-CD45 (#ab23910, Abcam, Cambridge, UK). After incubation with the appropriate fluorescence-conjugated secondary antibodies (1:1000 dilution, Alex Fluor 546 and 488, respectively; Invitrogen, Carlsbad, CA, USA), images were acquired using an inverted epi-fluorescence microscope (Olympus BX61) equipped with a DP72 camera.

### 2.3 Human saphenous veins culture ex vivo

HSVs, not required for surgery, were collected from discards after coronary artery bypass graft surgery and were cultured as previously described [3]. Briefly, the vein segments were opened longitudinally and cultured individually with luminal surface facing up in 12-well plates in RPMI 1640 medium supplemented with 30% FBS, 2 mmol/l L-glutamine, 100 IU/ml penicillin and 100 μg/ml streptomycin for 7 days with or without LP17 or TREM-1-activating antibody. The vein segments were then fixed and embedded in paraffin. Cross-sections were cut and stained with α-smooth muscle actin. Thickness of intima, media, and adventitia was measured by Image-Pro Plus 6.0.

### 2.4 Statistics

Data are expressed as mean±SD or SEM from 4 to 7 independent experiments. Differences between two groups were analyzed by two-tailed Student's t-test. Probability values less than 0.05 were considered statistically significant. All statistical analysis was performed with SPSS 19.0 for Windows (SPSS, Inc., Chicago, IL, USA).
